# Impact of fear on pediatric pain assessment: A retrospective study

**DOI:** 10.1371/journal.pone.0320995

**Published:** 2025-09-22

**Authors:** Myriam Aïche, Jean Toniolo, Emilie Auditeau, Stéphanie Thurillet, Laurent Fourcade

**Affiliations:** 1 CHU de Limoges, Hôpital de la Femme, de la Mère et de l’Enfant, Limoges, France; 2 Inserm, Université de Limoges, CHU Limoges, EpiMaCT - Epidemiology of chronic diseases in tropical zone, Institute of Epidemiology and Tropical Neurology, OmegaHealth, Limoges, France; 3 Département Universitaire de Sciences Infirmières, Faculté de Médecine et Pharmacie, Université de Limoges, France; CHU Nantes: Centre Hospitalier Universitaire de Nantes, FRANCE

## Abstract

**Background:**

Fear and pain are intimately linked in the experience of caring for a child. The advent of a self-assessment tool for fear makes it possible to study the link between these two emotions.

**Objectives:**

The primary objective was to determine whether self-assessment of fear (with the Trouillomètre^®^) affects self-assessment of pain. The three secondary objectives were to search for a correlation between fear and pain scores, and to analyze the effect on analgesic consumption and tool satisfaction.

**Methods:**

A retrospective study was carried out in a French university hospital comparing the VAS (visual analogue scale) values of children aged 7–12 presenting to pediatric ED before and after the introduction of a fear assessment by Trouillomètre^®^. Selected pain scores were measured using the VAS. Analgesic consumption was compared between the exposed and unexposed periods. Satisfaction was assessed using a questionnaire distributed to the paramedical team.

**Results:**

154 patients in the Trouillomètre^®^ group and 154 in the unexposed group were included in the analysis. There was no significant difference between the 2 groups in terms of pain assessment, but there was a significant correlation between fear and pain (rho = 0.18, p-Value = 0.002). Following the implementation of the tool, there was an increase in level 1 analgesic use and a corresponding decrease in level 2 analgesic use. The paramedical teams were satisfied with the tool.

**Conclusion:**

This study did not show any impact of fear assessment on pain assessment, but secondary analyses suggest that there is an effect of fear management on pain already described in the literature, and that Trouillomètre^®^ can be used to highlight this fear for its better management.

## Introduction

The experiences of hospitalized patients are closely linked to their emotional journey. In children, this complex element is composed of three interrelated notions: Fear, Pain and Anxiety [1]. The link between anxiety and pain has been studied in children in the past, with a particular focus on the potentiating effect of anxiety on pain experiences [2]. However, fear remains insufficiently explored and this gap is particularly noticeable in real-life clinical environments such as pediatric emergency departments, where time constraints, acute distress, and limited communication windows make it difficult to distinguish fear from pain, and yet critical treatment decisions depend on this distinction.

The neuroanatomical pathways involved in fear and pain utilize neural circuits that pass through common structures. In humans, the amygdala, located in the medial temporal lobe, plays a pivotal role in the learning and expression of conditioned fear responses [3]. In the traditional model of fear conditioning, it interacts with a set of structures involved in generating autonomic and behavioral responses to fear, such as immobilization.

Along the pathway to higher pain centers, synaptic contacts are established through collateral projections in various brainstem regions, notably the periaqueductal gray matter and the raphe nuclei, which play an essential role in the modulation of pain but also, as previously described, in the modulation of fear [4].

Functional imaging studies have also shown that spatial information related to nociceptive stimuli is processed in the amygdala, hippocampal complex, putamen, red nucleus, and cerebellum—structures that also contribute to guiding the defense cascade and withdrawal movement. [5,6] The biological interplay between fear and pain extends beyond anatomical structures, and the same hormones are secreted in response to both fear and pain stimuli [4,5].

Over the past years, pain management has emerged as a pivotal concern [7] and a central component of clinical practice in pediatric emergency departments. Effective pain management necessitates standardized assessment protocols, with analgesic doses being tailored according to the intensity of pain as reported by the patient (self-assessment) or in cases where the child is unable to provide self-reports by the caregiver (hetero-evaluation). Given that pain is inherently a subjective experience, self-report measures are regarded as the gold standard for pain assessment and should be utilized whenever feasible [8]. However, these pain scales are not specifically designed to assess fear, making it challenging to differentiate between these two experiences in the absence of reliable self-assessment tools [9]. Inappropriate—because subjective—interpretations of what children may be experiencing could lead to either inadequate pain management or, conversely, an overestimation of pain. For instance, a child’s fearful reactions during a medical procedure might be misinterpreted as severe pain, potentially resulting in unnecessary administration of stronger analgesics such as opioids. Indeed, peri‑operative pain management guidelines warn that misreading behavioral pain scores without accounting for emotional distress may lead to both under- and over‑use of analgesics in pediatric patients. [10]

To address this issue, the pediatric emergency department at Limoges University Hospital Center has developed and implemented a validated self-assessment tool for evaluating fear in children aged 7 to 12 years: the Trouillomètre^®^ [11]. This tool can be used to specifically identify and emphasize the fear component of the patient’s experience, thereby guiding the implementation of appropriate interventions. Given the close association between fear and pain, fear should not be overlooked in the management of pediatric patients. Several strategies can be used to address this issue, with distraction techniques playing a central role [12]. These may include activities such as games, parental involvement, virtual reality,... [13–15]. Several teams have attempted to address negative emotional responses (like anxiety) in pediatric emergency settings, notably by introducing interventions such as therapy dogs [16,17].

The primary objective of this study was to examine whether prior self-assessment of fear using the Trouillomètre® was associated with a reduction in self-reported pain scores (VAS) in children aged 7–12 years.

The secondary objectives were to assess the correlation between fear and pain scores, to evaluate the impact on analgesic consumption levels, and to evaluate the acceptability of the fear self-assessment tool among paramedical staff using the French System Usability Scale (F-SUS).

## Materials and methods

### Study design and setting

This monocentric retrospective cohort study was conducted in the pediatric emergency department of the Limoges University Hospital Center, France. It is reported in accordance with the STROBE guidelines.

### Study population and group allocation

We compared two groups of children aged 7 to 12 years who presented to the emergency department during two distinct time periods: an unexposed group, seen before the implementation of the Trouillomètre® tool, and an exposed group, seen after its integration into routine clinical care. In both groups, no fear-management interventions were performed prior to pain assessment, to minimize potential confounding in the measurement of pain scores.

### Data collection

Data collection for the unexposed group was conducted retrospectively using cases from 2022. For the exposed group, data were collected between February 21 and June 30, 2023, following the department-wide deployment of the Trouillomètre®. All data were extracted anonymously from the pediatric emergency department’s electronic medical record system (URQUAL) on June 30, 2023. Authors did not have access to any information that could identify individual participants during or after data collection.

### Inclusion and exclusion criteria

Inclusion criteria were defined as any child aged 7 to 12 years presenting to the pediatric emergency department during the study periods. Children below 7 or above 12 years of age were excluded because the Trouillomètre® tool has only been validated for use within this specific age range. Additional exclusion criteria included the presence of psychomotor disorders, significant language barriers, immediate need for surgical or resuscitative interventions, the use of pain scales other than the Visual Analogue Scale (VAS) for pain assessment, and formal opposition to the use of their data as recorded in the hospital’s data opt-out registry.

### Assessment tools and clinical procedures

In both groups, pain scores were documented using the VAS [18], as per departmental guidelines. Children were asked to move a cursor along a continuous line to indicate their perceived level of pain intensity. In the exposed group, fear levels were also assessed prior to pain assessment using the Trouillomètre® [11], which required the child to select the facial expression best representing their fear, prompted by a standardized verbal instruction. Pain management decisions were made only after both assessments had been completed. Analgesics were administered, if needed, according to institutional protocol: VAS scores between 1 and 3 warranted level 1 analgesics, scores from 4 to 7 warranted level 2, and scores from 8 to 10 led to the administration of level 3 analgesics.

### Outcome measures

The primary outcome was the comparison of mean pain scores (VAS) between the exposed and unexposed groups.

The secondary outcomes were the correlation between fear and pain scores in the exposed group, the comparison of analgesic consumption levels (according to the three VAS thresholds used in routine practice), and the usability score of the Trouillomètre® tool based on the French version of the System Usability Scale (F-SUS). [19]

### Usability evaluation

Tool usability was evaluated through the French version of the System Usability Scale (F-SUS) [19], which was adapted for this study by substituting the term “system” with “Trouillomètre®” in all items. The entire paramedical team of the pediatric emergency department completed the questionnaire anonymously, and two independent external evaluators conducted the analysis. According to established interpretive benchmarks, a score equal to or above 85 on the SUS is considered excellent, scores between 70 and 84 are considered good, and scores below 70 reflect room for improvement [20].

### Sample size and randomization

The required sample size was calculated prior to the study, based on the hypothesis that a clinically significant difference in VAS scores would be at least 0.84 points (as identified in the literature [21]). Assuming an alpha risk of 5% and a beta risk of 20%, the necessary sample size was estimated at 154 participants per group. Eligible patients were randomly selected using a computer-generated random number table.

### Statistical analysis

All data were processed and analyzed using SPSS version 22.0. Descriptive statistics were used to summarize the data, with continuous variables expressed as means and standard deviations, and categorical variables as frequencies and percentages. Normality of distribution was assessed using the Shapiro-Wilk test. The Mann-Whitney U test was used to compare continuous variables between the two groups, and the Chi-square test was used for categorical data. To identify the independent factors associated with the usability rating of the Trouillomètre®, a stepwise backward logistic regression analysis was performed, including all variables with a p-value less than 0.25 in univariate analysis. Collinearity between variables was assessed prior to model inclusion.

### Confounding factors and data integrity

Because age could influence both fear and pain perceptions, it was considered a potential confounding factor and included in adjusted analyses. Missing data were minimized through the mandatory documentation of both fear and pain scores in the electronic medical records. Patient records with missing values for either fear or pain assessment were excluded from the analyses to avoid classification bias.

### Ethical considerations

This study received approval from the Ethics Committee of the Limoges University Hospital Center (Approval No. 18-2023-03). All data used were fully anonymized, and the study complied with French and institutional data protection regulations.

## Results

**Fig 1:**
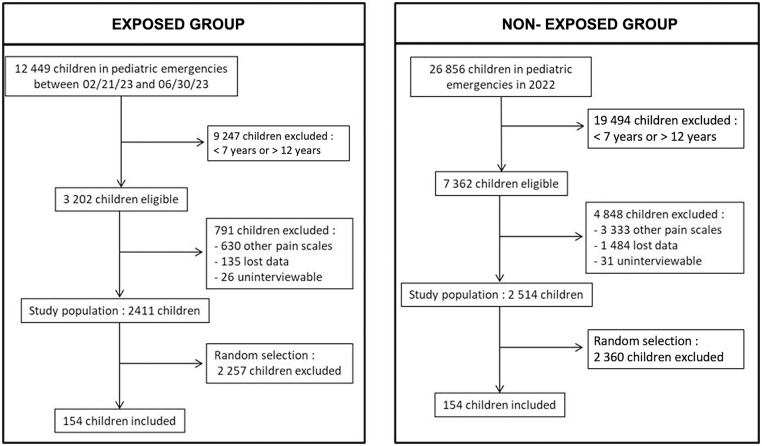
Flow chart. Flowchart of subject eligibility according to inclusion and exclusion criteria.

During the first period (non-exposed group: 2022), 7362 children aged 7 to 12 visited the pediatric emergency department of the CHU de Limoges. 4848 files were withdrawn from the analysis (other pain scale used, missing data, child unable to be interviewed). Ultimately, 2514 children were eligible. During the 2nd period (exposed group: 02/21/2023 to 06/30/2023), 3202 children aged 7 to 12 visited the pediatric emergency department of the CHU de Limoges. 791 files were withdrawn from the analysis (other pain scale used, missing data, child unable to be interviewed). Ultimately, 2411 eligible children visited the pediatric emergency department; a flowchart detailing the constitution of the two groups is shown in Fig 1. The characteristics of the two populations were similar, with the exception of age (Table 1).

**Table 1:** Demographic and Clinical Characteristics of Children Exposed or Not Exposed to the Fear Self-Assessment Tool (Trouillomètre®).

Variables		All (N = 308)	Before Trouillomètre^®^ Non-exposed (N = 154)	After Trouillomètre^®^ Exposed (N = 154)	p-value (test)
Sex	Female	138 (44.8%)	78 (56.5%)	60 (43.5%)	0.051 (X^2^)
	Male	170 (55.2%)	76 (44.7%)	94 (55.3%)	
Age (years)		9.9 ± 1.6	10.3 ± 1,6	9.53 ± 1.5	**< 0.001** (MW)
Reason for visiting (Missing data: n = 7)	Medical emergency	102 (49.8%)	55 (53.9)	47 (46.1%)	0.397 (X^2^)
	Trauma emergency	199 (66.1%)	96 (48.2%)	103 (51.8%)	

MW: Mann-Whitney test; X^2^: Chi-Squared test.

### Primary objective

The mean VAS score in the Trouillomètre^®^ group was 3.29 ± 2.7, while the mean VAS score in the unexposed group was 2.83 ± 2.6 (*p* = 0.133). There were no significant differences between the 2 groups. In the subgroups formed according to analgesic levels, there was no difference in the proportion of VAS grades between the 2 populations (Table 2).

**Table 2:** Comparison of Pain Scores (VAS) Between Children Exposed and Not Exposed to the Fear Self-Assessment Tool (Trouillomètre®).

Variable		All (N = 308)	Before Trouillomètre^®^ Non-exposed (N = 154)	After Trouillomètre^®^ Exposed (N = 154)	p-value (Test)
VAS	Mean	3.0 ± 2.6	2.83 ± 2.6	3.29 ± 2.7	0.133 (MW)
	Median (Q1; Q3)	3.0 (0; 8.0)	3.0 (0; 4.0)	3.0 (0; 5.0)	
Low VAS (< 3/10)		177 (57.5%)	86 (55.8%)	91 (59.1%)	0.645 (X^2^)
Moderate VAS (3–7/10)		93 (30.2%)	47 (30.5%)	46 (29.9%)	1.000 (X^2^)
Strong VAS (> 7/10)		38 (12.3%)	21 (13.7%)	17 (11%)	0.604 (X^2^)

MW: Mann-Whitney test; X^2^: Chi-Squared test; VAS: Visual Analog Scale; Q1: 1st quartile; Q3: 3rd quartile.

### Secondary objectives

#### Fear-pain link.

To investigate the relationship between fear and pain scores, we performed multiple linear regression (Table 3). The fear score was found to be an independent determinant of the pain score, while age was not.

**Table 3:** Multivariate Linear Regression Analysis of Factors Associated with Pain Scores (VAS) in Children Aged 7–12.

	Univariate model (N = 308) β (95% IC)	p-value	Multivariate model β (95% IC)	p-value
Age	0.146 (0.06–0.432)	**0.01**	0.133 (−0.039–0.519)	0.091
Trouillomètre^®^score	0.249 (0.102–0.438)	**0.002**	0.250 (0.104–0.438)	**0.002**

**Fig 2:**
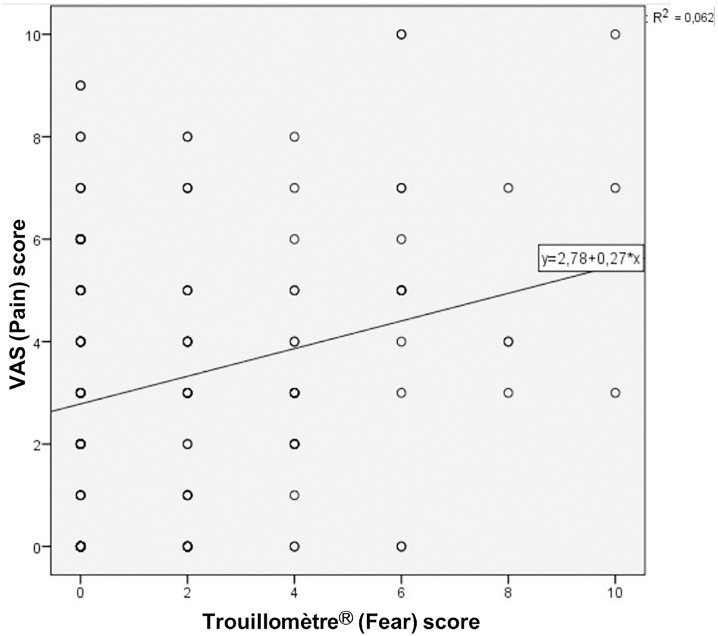
Linear regression. Linear regression curve showing the correlation between pain score (VAS) and fear assessment score (Trouillomètre^®^).

A statistically significant but weak correlation was observed between the pain score and the children’s fear score (rho = 0.18, p < 0.01; Fig 2).

#### Impact on analgesic consumption.

We assessed analgesic consumption by comparing analgesic consumption from March to June 2022 and March to June 2023 (Table 4). Consumption of level 1 analgesics increased significantly between 2022 and 2023, whereas consumption of level 2 analgesics decreased significantly. Consumption of level 3 analgesics and non-classifiable analgesics (Medical Nitrous Oxide and KETAMINE) did not change significantly.

**Table 4:** Comparison of Analgesic Consumption by WHO Analgesic Level Between 2023 and 2022.

	2022 (number of boxes)	2023 (number of boxes)	Movement	p-value (X^2^)
Level 1	3,192	3,902	+ 22.2%	**< 0,001**
Level 2	104	69	− 33.7%	
Level 3	166	192	+ 15.7%	0.407
Non-classifiable	80	83	− 3.8%	

X²: Chi-square test;

Level 1: Non-opioid analgesics (e.g., paracetamol, aspirin, NSAIDs);

Level 2: Weak opioids (e.g., codeine, tramadol);

Level 3: Strong opioids (e.g., morphine, oxycodone);

Non-classifiable: Agents not included in the WHO analgesic ladder (e.g., lidocaine, nitrous oxide/oxygen mixture).

### Tool feasibility in clinical practice

Thirty people (i.e., the entire pediatric ER paramedical team) completed the F-SUS questionnaire. One response was withdrawn because the questionnaire was not completed correctly. A score above 85.5 out of 100 is considered indicative of excellent acceptability. The mean score obtained for the Trouillomètre® was 88.44 ± 9.14, confirming a high level of acceptability among the paramedical staff.

## Discussion

This study investigated whether assessing fear prior to pain could influence subsequent pain scores in children presenting to the pediatric emergency department. Pain scores remained comparable regardless of whether fear was assessed first (mean VAS with fear-assessment: 3.29 ± 2.7 vs. without: 2.83 ± 2.6), suggesting no direct effect of fear assessment on pain perception. The absence of support for our hypothesis may be attributed to the study design: due to the need for procedural inconsistency and the lack of a standardized approach to fear management, pain assessment could not be conducted following fear management.

However, a significant correlation was observed between fear and pain (rho = 0.18), which supports existing neurophysiological and neuroendocrine evidence linking these two experiences [5,22–26]. Even though causality cannot be inferred from our data, this association invites further investigation into whether addressing fear may indirectly influence pain expression or perception. Although the strength of the correlation observed was weak, its statistical significance suggests a non-random relationship between the two variables. This modest effect size may be explained by the multifactorial nature of pain, which integrates biological, emotional, contextual, and cognitive dimensions beyond fear alone. Variability in emotional expression among children, the diversity of clinical presentations, and individual differences in pain thresholds may also dilute the strength of the association in a real-life emergency setting. Nevertheless, the identification of even a weak correlation supports the idea that fear contributes, at least in part, to the perception of pain and highlights the clinical relevance of integrating emotional assessment into pain management strategies. The observation of this correlation, despite the broad variability in reasons for consultation in pediatric emergency departments—from simple viral infections to open fractures—supports the notion of a strong connection between fear and pain. To our knowledge, there are no existing data in the literature that demonstrate this link in children, particularly in the absence of a reliable tool for assessing fear. Hedén et al. [27], alluded to this connection, but they used the same scale [28] to assess both fear and pain. Subjective analyses, particularly those from parents’ perspectives, have suggested the presence of this link [29]. Future development of fear assessment tools could include a parallel parent-report component—such as structured questionnaires or adapted visual analogue scales—to complement child self-reports and clinical observations. This finding encourages further exploration of concurrent management of both emotions. Although it is well established that pain scales are not suitable for assessing fear [9], the reverse has not been conclusively demonstrated. However, the psychometric properties of the Trouillomètre^®^ have confirmed that children significantly associate the faces on the scale with fear [11].

Interestingly, despite the absence of a significant difference in VAS scores, fear assessment led to changes in analgesic prescribing patterns, with reduced use of Level 2 analgesics (–33.7%) and increased use of Level 1 analgesics (+22.2%) in the intervention group. This finding aligns with prior literature indicating that fear modulation strategies (e.g., distraction, explanation) can reduce children’s perceived pain or anxiety [30–32]. It is plausible that by identifying and addressing fear more appropriately, caregivers were more confident in prescribing lower-intensity analgesics for some children who might otherwise have received stronger medications. This may contribute to minimizing unnecessary exposure to opioids or stronger medications in children.

Several methodological limitations could explain the absence of a stronger effect. The study design did not allow for standardized fear management following its assessment, which may have diluted the potential impact on pain. Furthermore, the timing of the fear assessment—conducted upon arrival—might not have captured the peak of emotional distress, as fear in pediatric emergencies often emerges during or after medical interventions [33,34]. Future studies should consider assessing fear at multiple time points during the child’s ED trajectory, particularly after potentially distressing procedures. Additionally, parental fear—known to influence children’s emotional states—was not accounted for in our analysis, though its inclusion in future studies may enhance our understanding of the fear–pain dynamic [35]. Finally, should standardized distraction interventions be successfully implemented, conducting a study to evaluate their effects would be of interest.

Initial sample size calculations were based on literature reporting average pain scores around 5/10 [36,37], aiming for a clinically meaningful reduction below the 3/10 threshold—consistent with our protocol for initiating Level 1 analgesia. However, our study population displayed lower baseline pain (VAS ≈ 3/10), suggesting that prior estimates (from non-European settings) were not representative. This raises questions about whether our predefined reduction target—based on higher baseline scores in earlier studies (VAS = 5.22/10)— was appropriate for our lower-scoring population. Therefore, we decided that the most clinically relevant approach would be to assess the impact on changes in pain levels. However, even among children with VAS > 5, we observed a trend toward lower scores in the fear-assessed group, though not statistically significant. This discrepancy raises questions about the appropriateness of our predefined threshold and highlights the need for localized baseline data in future study designs.

Another limitation was the lack of age matching between groups. However, age was not identified as an independent determinant of pain score, suggesting limited impact on our primary findings. Ideally, a randomized controlled trial would have ensured group comparability, but such a design was not feasible within the logistical constraints of our clinical setting.

Importantly, this study represents the first large-scale clinical deployment of the Trouillomètre®. Its integration into practice was straightforward: paramedical teams reported high usability scores (F-SUS above acceptability threshold), and data completeness exceeded 95%, indicating strong adherence. This also helps explain the numerical similarity between the two study populations, despite significant differences in the eligible populations. We instructed ED paramedical teams to prioritize the use of the VAS in children aged 7–12 years when appropriate, as we selected this scale to assess the impact of fear evaluation. This operational success, combined with the scale’s psychometric properties [11], supports its external validity. Notably, fear scores in our cohort were comparable to those from the initial pilot study (Trouillomètre® = 1.75 ± 2.62), reinforcing its reliability and reproducibility in real-world conditions.

Our findings highlight the value of incorporating fear assessment into routine pediatric emergency care—not only to better understand the interplay between emotion and pain, but also to inform more tailored and potentially less aggressive therapeutic approaches. Future development of complementary tools (e.g., parent-reported fear scales) and interventional studies targeting fear reduction could further optimize pediatric emergency care.

## Conclusion

Contrary to our initial hypothesis, the assessment of fear prior to pain evaluation did not directly reduce the reported pain scores. However, the availability of a dedicated tool to identify fear—especially in children who exhibited no obvious signs—enabled clinicians to recognize and often manage fear informally during routine care. Although this fear management was neither standardized nor formally assessed, it may have contributed to enhanced patient comfort and, indirectly, to a reduced need for higher-level analgesics.

Since analgesic administration typically occurs after both fear assessment and any informal intervention, our findings support the hypothesis that recognizing and addressing fear may influence how children experience and report pain. In addition to supporting existing neurobiological data, our study is the first to quantify the relationship between fear and pain in children using a validated self-assessment tool. Finally, the integration of this additional step into the clinical workflow was well accepted by paramedical teams, reinforcing the practical value of the tool in real-world settings.
